# PD-L1 amplification is associated with an immune cell rich phenotype in squamous cell cancer of the lung

**DOI:** 10.1007/s00262-020-02825-z

**Published:** 2021-02-12

**Authors:** Torsten Goldmann, Sebastian Marwitz, Dörte Nitschkowski, Rosemarie Krupar, Max Backman, Hedvig Elfving, Viktoria Thurfjell, Amanda Lindberg, Hans Brunnström, Linnea La Fleur, Artur Mezheyeuski, Johanna Sofia Margareta Mattsson, Johan Botling, Patrick Micke, Carina Strell

**Affiliations:** 1 Division of Pathology, Research Center Borstel, Leibniz Lung Center, Borstel, Germany; 2 Airway Research Center North (ARCN), Member of the German Center for Lung Research (DZL), Großhansdorf, Germany; 3 Institute of Pathology, University Hospital Schleswig-Holstein, Campus Lübeck, Lübeck, Germany; 4 Department of Immunology, Genetics, and Pathology, Uppsala University, Dag Hammarskjölds väg 20, 751 85 Uppsala, Sweden; 5 Division of Pathology, Department of Clinical Sciences Lund, Lund University, Lund, Sweden; 6 Division of Laboratory Medicine, Department of Genetics and Pathology, Region Skåne, Lund, Sweden

**Keywords:** Check-point inhibitors, Lung cancer, Microenvironment, Immunotherapy, PD-L1 amplification

## Abstract

Gene amplification is considered to be one responsible cause for upregulation of Programmed Death Ligand-1 (PD-L1) in non-small cell lung cancer (NSCLC) and to represent a specific molecular subgroup possibly associated with immunotherapy response. Our aim was to analyze the frequency of PD-L1 amplification, its relation to PD-L1 mRNA and protein expression, and to characterize the immune microenvironment of amplified cases. The study was based on two independent NSCLC cohorts, including 354 and 349 cases, respectively. Tissue microarrays were used to evaluate PD-L1 amplification by FISH and PD-L1 protein by immunohistochemistry. Immune infiltrates were characterized immunohistochemically by a panel of immune markers (CD3, CD4, CD8, PD-1, Foxp3, CD20, CD138, CD168, CD45RO, NKp46). Mutational status was determined by targeted sequencing. RNAseq data was available for 197 patients. PD-L1 amplification was detected in 4.5% of all evaluable cases. PD-L1 amplification correlated only weakly with mRNA and protein expression. About  37% of amplified cases were negative for PD-L1 protein. PD-L1 amplification did not show any association with the mutational status. In squamous cell cancer, PD-L1 amplified cases were enriched among patients with high tumoral immune cell infiltration and showed gene expression profiles related to immune exhaustion. In conclusion, PD-L1 amplification correlates with PD-L1 expression in squamous cell cancer and was associated with an immune cell rich tumor phenotype. The correlative findings help to understand the role of PD-L1 amplification as an important immune escape mechanism in NSCLC and suggest the need to further evaluate PD-L1 amplification as predictive biomarker for checkpoint inhibitor therapy.

**Supplementary Information:**

The online version contains supplementary material available at 10.1007/s00262-020-02825-z.

## Introduction

Checkpoint inhibitors have recently become approved as a promising therapy option in patients with advanced non-small cell lung cancer (NSCLC). However, only a minority of patients experience a long-term, durable, clinical response. To enrich the patient population that benefits from immunotherapy, several biomarkers have been suggested. Of them, PD-L1 expression of tumor cells, determined by immunohistochemistry, is most established and serves as an approved diagnostic biomarker for most indications in the first- and second-line treatments of NSCLC [1, 2]. Although assessment of PD-L1 expression increases the number of responders, the predictive accuracy is only modest [3].

PD-L1 protein expression by cancer cells is an effective immune evasion mechanism in the progression of cancer. Several studies suggest that one important mechanism of PD-L1 up-regulation is based on gene amplification ([4], reviewed in Ref. [5]). It can be speculated that a genomic aberration leads to a higher, homogenous, and more stable upregulation of PD-L1 compared to other regulative mechanisms. The inhibitory effect on the immune microenvironment might be stronger and connected to a growth advantage for PD-L1 amplified cancer cells [6]. Indeed, studies on many cancer forms have identified the chromosomal region 9p24.1 to be amplified, which includes the gene locus for PD-L1 [7–10]. A comprehensive analysis of TCGA-data sets showed that the frequency of amplification was between 2 and 10%, depending on the tumor type [11]; moreover, PD-L1 amplification has also been described in NSCLC. The first study demonstrated a PD-L1 amplification in 5% of the NSCLC cases and that copy number gain was associated with a higher PD-L1 protein expression [12]. The proportion of PD-L1 amplification and the strong association with PD-L1 protein expression has been confirmed in independent NSCLC cohorts [13–15]. The clinical relevance of this genomic aberration is indicated by the observation that NSCLC patients with PD-L1 amplified tumors have a worse prognosis [15].

With this background, the aim of our study was to use integrative analysis to characterize PD-L1 amplification, PD-L1 gene and protein expression in NSCLC, and based on the clinico-pathological and molecular background define the immune phenotype that is associated with an increased PD-L1 gene copy number.

## Materials and methods

### Patient cohorts and cancer tissues

The cohorts consisted of patients who underwent an operation at the Uppsala University hospital (Uppsala, Sweden) between 1995 and 2005 (Uppsala 95, *n* = 349) and between 2006 and 2010 (Uppsala 06, *n* = 354). The characteristics of both cohorts are described in Table 1. Two tissue cores from each patient had been incorporated into the tissue-microarrays (TMAs), as described previously [16–19]. Information on clinical parameters were obtained from the records of the population based Uppsala-Örebro Regional Lung Cancer Register and from patient records. The study was conducted in accordance with the Declaration of Helsinki and the Swedish Ethical Review Act (Ethical Review Board in Uppsala, Uppsala 95: #2006/325, Uppsala 06: #2012/532).

**Table 1:** Clinical data and patient characteristics Abbreviations: WHO = world health organization; AC = adenocarcinoma; SqCC = squamous cell carcinoma; AdSq = adenosquamous carcinoma; LCC = large cell carcinoma; LCNEC = large cell neuroendocrine carcinoma; SC = sarcomatoid carcinoma

	Uppsala 06	Uppsala 95
Total	354	349
Included in study (PD-L1 FISH data available)	310	224
Age, years		
≤ 70	195 (62.9)	154 (68.8)
> 70	115 (37.1)	70 (31.2)
Gender		
Female	159 (51.3)	102 (45.5)
Male	151 (48.7)	122 (54.5)
Stage		
Ia–Ib	195 (62.9)	158 (70.5)
Iia–IV	115 (37.1)	66 (29.5)
WHO performance score		
0	193 (62.3)	116 (51.8)
1–3	117 (37.7)	107 (47.8)
4	–	1 (0.4)
Smoker		
Never	34 (11)	19 (8.5)
Former or current	276 (89)	203 (90.7)
Missing	–	2 (0.8)
Histology		
AC	196 (63.2)	119 (53.1)
SqCC	94 (30.3)	82 (36.6)
AdSq	5 (1.6)	–
LCC	5 (1.6)	23 (10.3)
LCNEC	8 (2.6)	–
SC	2 (0.7)	–
Data available for		
PD-L1 IHC	303/310	207/224
PD-L1 mRNA	180/310	122/224

### Fluorescence in situ hybridization (FISH)

FISH was performed on the TMAs of both cohorts as described previously [12]. A dual color probe for CD274 and the classical satellite III region of chromosome 9 (D9Z3) (Zytolight SPEC CD274, PDCD1LG2/CEN9, Zytovision, Germany) were used according to the manufacturer’s instructions. The probe consists of a ZyGreen labeled probe, specific for CD274 at 9p24.1 and ZyOrange labeled CEN9 probe, specific for D9Z3 at 9q12; nuclei were stained with DAPI. FISH was evaluated using a Nikon eclipse 80i microscope equipped with a Plan Apo VC 60 × lens using oil immersion, by two independent observers. Images were taken by a Leica DFC 450c camera system and Fix Foto software (Joachim Koopmann Software, Germany). Images were adjusted for brightness and contrast. Single fluorescence images (blue, green, and orange) were taken and overlayed. Cases were considered as being PD-L1 amplified, when the ratios of PD-L1 signals to centromere 9 signals in the tumor cells were 2 or lager. Cases were defined as copy number gain due to polysomy when the ratios of PD-L1/centromere 9 were 1 but there were more than two copies of PD-L1 and centromere 9 within a single tumor cell (Supplementary Fig. 1).

### Immunohistochemistry

Immunohistochemistry (IHC) of 4-μm thick TMA sections was performed as previously described [20]. The full protocol is available at the website of the human protein atlas (ref. http://www.proteinatlas.org/download/IHC_protocol.pdf). The antibodies used for the IHC analyses were the following: CD3ε (Atlas Antibodies, CL1497; 1:1000 dilution), CD4 (Atlas Antibodies, CL0395; 1:125 dilution), CD8A (Atlas Antibodies, CL1529; 1:250 dilution), CD20 (Dako, L26, pre-prepared manufacturer dilution), CD45RO (Abcam, UCHL1, 1:1000 dilution), CD138 (Dako, MI15, 1:100 dilution), CD163 (Novocastra, 10D6, 1:100 dilution), FOXP3 (Santa Cruz Biotechnology, 236A/E7, 1:15 dilution), PD-1 (Abcam, AB52587, 1:200 dilution), and natural killer (NK)p46 (R&D systems, 195,314, 1:50 dilution). PD-L1 (Agilent, 22C3, pre-prepared dilution) staining was performed at the clinical pathology unit at Uppsala University Hospital on a DAKO autostainer system following the manufacturer’s instruction including antigen retrieval at pH6.

Immune marker–positive cells were visually annotated as a percentage of stained nucleated cells in the respective stroma and tumor compartment for the whole tissue area of both TMA cores. The immune cell score in the stroma compartment was calculated by dividing the positive immune cells by all immune cells and all other stroma cells (fibroblasts, endothelial cells, etc.). In the tumor compartment, the immune score was calculated by dividing the positive immune cells by all cells (tumor cells and immune cells). The increments used for visual annotation were 0%, 1%, 5%, 10%, 15%, 20%, 25%, 30%, 35%, 40%, 50%, 60%, 70%, 80%, 90%, and 100%. The final overall immune score per patient was calculated as the average of the stroma and tumor compartment immune scores. For the annotation of PD-L1 staining in the tumor compartment, we used the common annotation of cancer cell staining, used in clinical diagnostics (tumor proportion score), which is the percentage of viable tumor cells showing a partial or complete membrane staining (same percentage increments as above).

### Gene expression

The Uppsala 06 cohort was analyzed by RNA sequencing. Corresponding gene expression data were available for 197 patients, obtained from RNA-sequencing as previously described [21]. RNA was extracted from fresh frozen tissue and prepared for sequencing using Illumina TruSeq RNA Sample Prep Kit v2 with polyA selection. The sequencing was performed based on the standard Illumina RNAseq protocol with a read length of 2 × 100 bases. The raw data together with clinical information are available on the gene expression omnibus, with the accession number GSE81089.

Gene expression in the validation cohort Uppsala 95 was analyzed by microarrays. RNA from frozen tumor tissue from 193 patient samples included in the Uppsala 95 cohort were used for gene expression microarray analysis with the Affymetrix HG U133 Plus 2.0 arrays (54,675 probe sets, Affymetrix, Santa Clara, CA, U.S.A.), as described previously [18]. The microarray data set is deposited in the Gene Expression Omnibus data repository with the accession number GSE37745. For PD-L1, two probe sets (223834_at and 227458_at) were included on the Affymetrix chip, and the average expression level was used in the analyses.

### Mutation analysis

Target enrichment and deep sequencing were performed for 352 patients of the Uppsala 06 cohort using the HaloPlex Target Enrichment System (Agilent Technologies, Santa Clara, U.S.A.) including all coding exons of 82 lung cancer related genes, as previously described [22]. The tumor mutational burden was estimated by dividing the number of synonymous and non-synonymous mutations in a sample by the size of the sequenced genome (0.47 Mb). For the *KRAS, EGFR, PIK3CA, NRAS, BRAF, ERBB2,* and *MET* genes, only the activating driver mutations were considered in the analysis.

### Statistical analysis

The analyses were restricted to adenocarcinoma (AC) and squamous cell carcinoma (SqCC) cases, corresponding to the WHO classification from 2015 [23]. In total, 290 cases could be evaluated for PD-L1 amplification (196 AC and 94 SqCC cases, Table 1). Hazard ratios (HR) with 95% confidence interval (CI) were calculated with Cox proportional hazards regression in SPSS (IBM SPSS Statistics version 25). The differences between the two independent groups were compared using Mann–Whitney *U* test and in case of more than two groups by Kruskal–Wallis test with Dunn’s correction for multiple testing. Associations between PD-L1 amplification status and clinical parameters were evaluated by Fisher’s exact test. All statistical tests were two-sided, and *p* < 0.05 was considered statistically significant.

Hierarchical clustering was performed using the *pheatmap* package in R (R Studio, Version 1.2.5001) with Ward’s (Ward.D2) method and Euclidean distance. Linear normalization (0 to 1 range) was applied to the immune scores of each marker for cluster analysis. Differential gene expression analysis and gene ontology were done using raw count sequencing data with the *DESeq2* package in R, including false discovery rate (FDR) based adjustment of p-values using the Benjamin-Hochberg procedure. Genes with a FDR < 0.05 were included in gene ontology analysis using the R package TopGO, with all expressed genes of our dataset as a reference. Enrichment was tested with Fisher’s exact test. Only ontology terms comprised of > 10 genes were considered.

## Results

### Frequency of PD-L1 amplification and association with clinico-pathological parameters

**Fig. 1:**
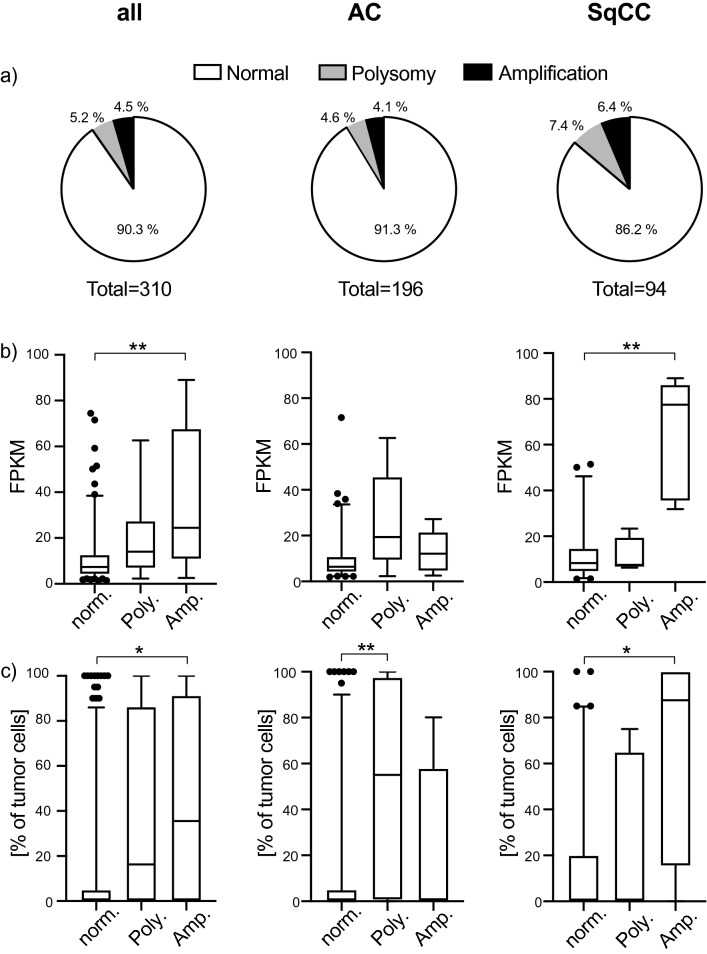
**a** Percentual distribution of the PD-L1 amplified cases and cases with PD-L1 polysomy among all included NSCLC patients of the Uppsala 06 cohort as well as separately within the adenocarcinoma (AC) and squamouscell carcinoma (SqCC) tumor subgroups. **b**, **c** Association of the PD-L1 amplification status with (**b**) PD-L1 mRNA expression (as FPKM) and (**c**) percentage of PD-L-positive tumor cells on protein level as determined by IHC. Box plots indicate the median with the interquartile range and the whiskers represent the 5–95 percentiles. Outliners are represented as dots. *p* values are based on Kruskal–Wallis test (two-sided) with Dunn's correction for multiple testing. *p < 0.05; **p < 0.001

The PD-L1 amplification (PD-L1/centromere 9 ratio of ≥ twofold) was analyzed by FISH (Supplementary Fig. 1) in TMAs of the NSCLC Uppsala 06 and Uppsala 95 cohort. Clinical data and patient characteristics for both cohorts are presented in Table 1. In the Uppsala 06 cohort, 14 out of the 310 (4.5%) evaluable patients showed PD-L1 amplification (Fig. 1a). An additional 16 (5.2%) cases demonstrated a PD-L1 gene copy number gain based on polysomy of chromosome 9. The PD-L1 amplification as well as the polysomy were slightly more frequent in squamous cell carcinomas (SqCC) cases than in the adenocarcinomas (AC) cases (Fig. 1a).

Within the Uppsala 95 cohort, PD-L1 amplification was detected in 10 (4.5%) and polysomy in 3 (1.3%) out of the 224 evaluable NSCLC cases (Supplementary Fig. 2A). The frequency of the PD-L1 amplification or polysomy was marginally higher in the SqCC cases than in the AC cases (Supplementary Fig. 2A).

No association was seen between the PD-L1 amplification and other clinico-pathological parameters for both cohorts (Supplementary Table 1), including sex, age, stage, and performance status. The only exception was in the Uppsala 95 cohort, showing a higher PD-L1 amplification frequency for the large cell carcinoma histological subtype (*p* = 0.021, Fisher’s exact test). Survival analysis did not reveal any association with either the PD-L1 amplification or the polysomy in uni- and multivariable Cox regression analysis in both cohorts (Supplementary Table 2).

### PD-L1 amplification, gene expression, and protein expression

In the Uppsala 06 cohort, PD-L1 amplification correlated with PD-L1 mRNA expression (RNA sequencing-based; median FPKM value in the non-amplified group 7.3 versus 24.4 in the PD-L1 amplification group, *p* = 0.005 Kruskal–Wallis test, Fig. 1b) as well as PD-L1 protein levels in tumor cells (IHC-based; median positive tumor cells 0% versus 35.5%, *p* = 0.043; Fig. 1c). Furthermore, the degree of PD-L1 amplification correlated with mRNA (*r* = 0.23, *p* = 0.002 Spearman’s rank test) and protein expression (*r* = 0.14, *p* = 0.018) (Supplementary Fig. 3). Of note, 5 out of 14 amplified cases (35.7%) were negative for PD-L1 protein (≤ 1%) in tumor cells. In contrast, the majority (74%) of strongly positive cases on protein level (PD-L1 > 50%) were neither amplified nor displayed polysomy. Subgroup analysis revealed that the association of PD-L1 amplification with PD-L1 mRNA and protein level was restricted to the SqCC subtype (mRNA *p* = 0.002, protein *p* = 0.022, Kruskal–Wallis test), while in AC, the correlation remained significant only on protein level and only for those cases exhibiting polysomy (*p* = 0.007) (Fig.1b,c).

The positive association between PD-L1 amplification and PD-L1 protein levels in tumor cells (median positive tumor cells 0% in the non-amplified group versus 15% in the PD-L1 amplification group, *p* < 0.001) (Supplementary Fig. 2C) as well as the correlation between the degree of PD-L1 amplification and protein level (*r* = 0.26, *p *< 0.001) (Supplementary Fig. 3) were confirmed in the Uppsala 95 cohort. Again, it was noted that 75% of the cases with more than 50% PD-L1 protein positive tumor cells were neither amplified nor displayed polysomy and vice versa, 4 out of the 10 PD-L1 amplified cases (40%) were negative for PD-L1 protein. No statistically significant correlations between the PD-L1 amplification and mRNA status, as based on Affymetrix array analysis, could be demonstrated when the complete cohort was analyzed. Statistical analyses of the AC and SqCC subgroups in the Uppsala 95 cohort were not feasible because of the low case numbers (Supplementary Fig. 2B and C).

Since polysomy in general did not demonstrate a consistent correlation with PD-L1 gene and protein expression in the complete cohort, we focused on PD-L1 amplified cases in the further analysis.

### PD-L1 amplification is not associated with the tumoral mutation status

**Fig. 2:**
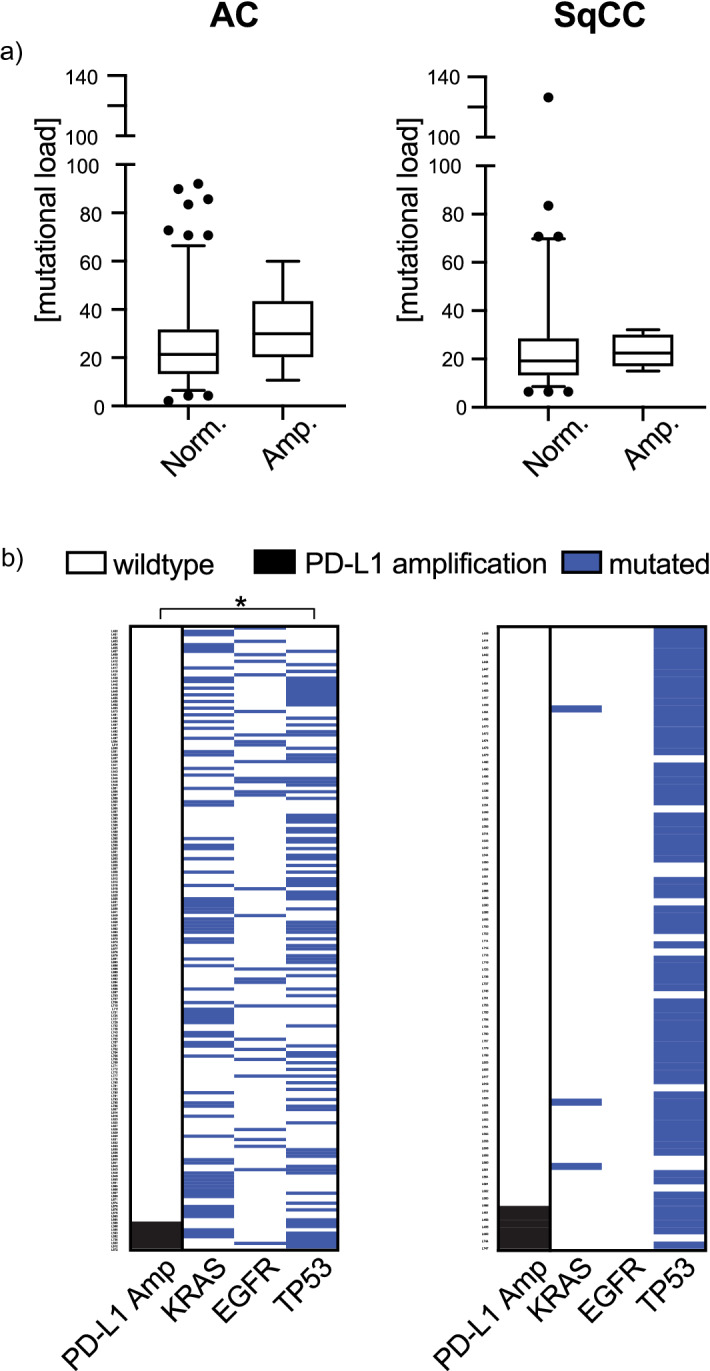
**a** Association of the PD-L1 amplification status with mutational load (synonymous plus non-synonymous) within the adenocarcinoma (AC) and squamouscell carcinoma (SqCC) tumor subgroups of the Uppsala 06 cohort. Box plots indicate the median with the interquartile range and the whiskers represent the 5–95 percentiles. Outliners are represented as dots. **b** Association of the PD-L1 amplification status (black) with activating driver mutations of the *EGFR* and *KRAS* genes as well as mutations of the *TP53 gene* (blue) are represented as binary heat maps. P-value is based on Fisher’s exact test, two-sided. *p < 0.05

The PD-L1 amplified cases of the Uppsala 06 cohort showed a statistically non-significant tendency toward a higher mutational load (*p* = 0.151 Mann–Whitney *U* test, Fig. 2a) in AC. When the most frequent mutations (*EGFR*, *KRAS,* or *TP53*) were analyzed, a significant association of PD-L1 amplification and *TP53* mutation was noted in the AC subgroup (*p* = 0.028, unadjusted Fisher’s exact test) (Fig. 2b). However, including all 82 mutations of genes, no statistically significant associations were detected between any of the mutations and the PD-L1 amplification status after adjustment for multiple testing (Supplementary Fig. 4).

### Relation between PD-L1 amplification and tumor immune cell infiltration

**Fig. 3:**
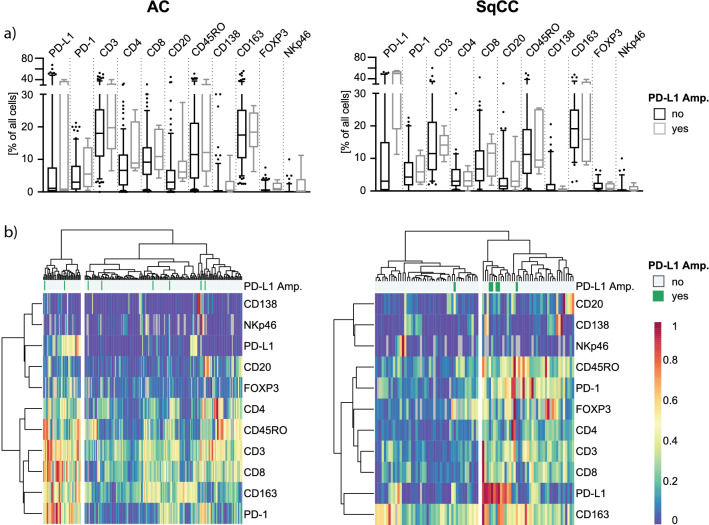
**a** Association of the PD-L1 amplification status with single immune cells counts within the adenocarcinoma (AC) and squamouscell carcinoma (SqCC) tumor subgroups of the Uppsala 06 cohort. Immune cells counts were determined by IHC. Box plots indicate the median with the interquartile range and the whiskers represent the 5–95 percentiles. Outliners are represented as dots. **b** Hierarchical cluster analysis using Ward’s method and Euclidian distance of the IHC based immune marker annotation and its association with PD-L1 amplification status and tumor histology

Single immune cell counts using antibodies against the markers PD-1, CD3, CD8, CD4, CD20, CD45RO, CD138, CD163, FOXP3, and NK46p were determined within the tumor and the associated stroma for the Uppsala 06 cohort. No statistically significant differences were noted in the distribution of the single immune cell markers between PD-L1 amplified and non-amplified cases (Fig. 3a).

Hierarchical cluster analysis was done to define the immune phenotypes for the AC and the SqCC subgroups. In both histological subtypes, two clusters were identified: one cluster characterized by an overall low immune cell infiltration referred to as “immune cell poor”, and one cluster characterized by an overall higher immune cell infiltration referred to as “immune cell rich.” It was only in the SqCC subgroup that PD-L1 amplified cases were enriched in the patient cluster displaying an immune cell rich patter, albeit not significant (*p* = 0.096, Fisher’s exact test) (Fig. 3b). This cluster was characterized by high numbers of PD-1, CD3, CD8, and CD45RO-defined T cells and CD163-defined macrophages, together with a high expression of PD-L1 protein. Accordingly, the Uppsala 95 cohort, as analyzed with a reduced immune marker set, showed that all 3 PD-L1 amplified cases were grouped in the immune cell rich cluster in SqCC (*p* = 0.103, Supplementary Fig. 5).

Additional analyses using the RNAseq data of the Uppsala 06 cohort indicated that in SqCC, the PD-L1 amplified cases were also enriched among samples with higher mRNA expression levels of marker genes for immune exhaustion as well as a higher expression of HLA genes (Supplementary Fig. 6).

### Differential gene expression analysis of PD-L1 amplified cases

**Fig. 4:**
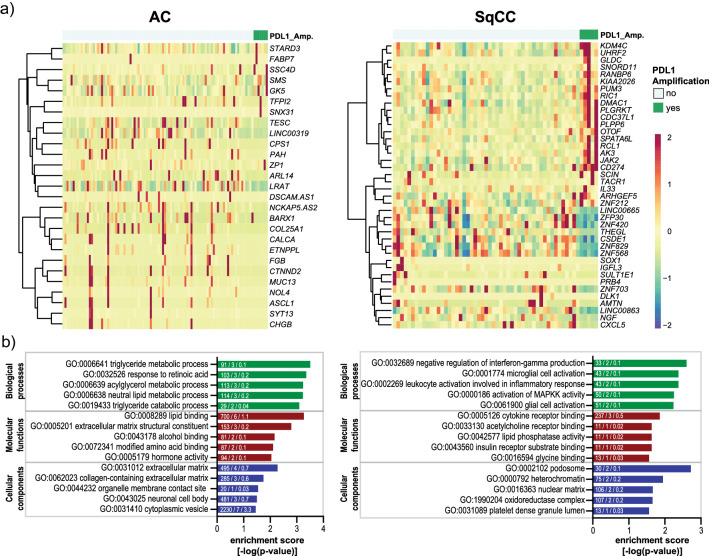
**a** Summary of genes differentially expressed in the PD-L1 amplified cases (FDR < 0.05) based on a separate analysis of the adenocarcinoma (AC) and squamous cell carcinoma (SqCC) tumor subtypes of the Uppsala 06 cohort. Genes were clustered hierarchically using Ward’s method and Euclidian distance. **b** Gene ontology (GO) analysis of PD-L1 amplified cases of AC and SqCC. Gene ontology analysis was performed on differentially expressed genes with *p*-adjusted < 0.05. Only GO terms comprised of > 10 genes were considered. The bars represent the corresponding enrichment scores. Enrichment scores represent the negative logarithm of the enrichment *p *value based on Fisher’s exact test. Numbers stated within the bars indicate the “number of annotated genes in GO term”/“number of genes significantly enriched”/and the “number of expected genes.”

Comparative analysis of RNAseq data between the PD-L1 amplified and non-amplified cases of the Uppsala 06 cohort revealed 27 statistically significant differentially expressed genes in the AC subgroup and 40 in the SqCC subgroup (FDR < 0.05) (Fig. 4a and Supplementary Tables 3 and 4). In the AC subgroup, the majority of these genes were down-regulated (Fig. 4a left panel and Supplementary Table 3), and gene ontology revealed a relation to metabolic processes and matrix remodeling (GO:0031012, GO:0062023) (Fig. 4b left panel). In the SqCC subgroup, most of the differentially expressed genes (*KDM4C, UHRF2, GLDC, RANBP6, KIAA2026, RIC1, JAK2, DMAC1, PLGRKT, CDC37L, PLPP6, SPATA6L, RCL1, AK3,* and *IL-33*) are located on 9p24.1 and accordingly showed an overexpression in the PD-L1 amplified cases (Fig. 4 right panel and Supplementary Table 4). This also included the oncogene Janus kinase 2 (*JAK2*), which is co-amplified with *CD274* (PD-L1) in classical Hodgkin lymphoma, triple negative breast cancer, and renal cell cancer as well as in NSCLC [9, 13, 14, 24]. The genes differentially expressed in the SqCC subgroup were amongst others connected to gene ontology terms of negative regulation of interferon-gamma production (GO:0032689), leukocyte activation involved in inflammatory response (GO:0002269), cytokine receptor binding (GO:0005126), and heterochromatin (GO:0000792) (Fig. 4b right panel).

## Discussion

Our study provides an in-depth analysis of PD-L1 amplification in NSCLC. We confirmed correlations of gene amplification with mRNA levels and protein expression that were more pronounced in the SqCC histological subgroup. Furthermore, we found that SqCC with PD-L1 amplified cases were enriched among tumors with an immune cell rich phenotype.

As previously described [12, 13, 15, 25], PD-L1 gene amplification showed a strong but not perfect association with PD-L1 gene and protein expression. The finding that the majority of strong PD-L1 protein positive cases (> 50% tumor proportion score) were not PD-L1 amplified and the existence of cases that were negative for PD-L1 protein within the PD-L1 amplified group indicates that there are other mechanisms to regulate PD-L1 protein expression, which are described for distinct genomic subgroups [5, 26, 27]. Also, promotor methylation, histone H3 acetylation, or microRNAs might play a regulative role, although this has not yet been demonstrated in clinical NSCLC patient samples [28–30]. In this respect, there might be differences between the histological subtypes. We found that the proportion of PD-L1 amplified cases was higher in the SqCC subgroup and that most amplified cases showed at least weakly positive PD-L1 protein expression in contrast to the AC subtype.

The higher proportion of PD-L1 amplification in SqCC was observed in two previous studies, where more than twice the number of PD-L1 amplified cases was observed in the SqCC subtype compared to the AC subtype [12, 15]. In our study, we observed an additional characteristic that might indicate that PD-L1 amplification has a biological impact, particularly in SqCC: Our hierarchical cluster analysis demonstrated that PD-L1 amplified SqCC clearly grouped within samples that had a general immune cell rich phenotype. PD-L1 amplified SqCC cases were further grouped within the samples, displaying a high expression of marker genes for immune exhaustion. These features together are considered to characterize cases that might be particularly sensitive to checkpoint inhibition [31], thus, indicating that PD-L1 amplification might be of clinical relevance.

Indeed, classical Hodgkin lymphoma is characterized by the chromosomal amplification of 9p24.1, including the gene locus for PD-L1. The overexpression of PD-L1 presents an effective immune evasion mechanism from the extensive immune reaction that is typical for this cancer type. Consequently, intervention with checkpoint inhibitors showed impressive long-term response rate between 60 and 90% in relapsed or refractory classical Hodgkin lymphoma [32].

In correspondence to Hodgkin lymphoma, our study showed that PD-L1 amplified SqCC cases are associated with an activated immune microenvironment with prominent immune cell infiltration. Surprisingly, since the first description of the presence of PD-L1 amplification in NSCLC [12], the effectiveness of checkpoint inhibitors for this particular patient group has not been evaluated so far [25]. Nevertheless, PD-L1 amplification might present a useful biomarker that in addition to PD-L1 immunohistochemistry could refine the selection of SqCC patients benefiting from immune checkpoint inhibitor therapy. Also, the issue of tissue heterogeneity would not be as crucial for a stable molecular aberration [33–35]. Finally, this genomic change can be easily included in next generation sequencing strategies in the diagnostic routine. The current genomic panels are already able to detect mutations and gene copy number variations simultaneously [36–38].

However, it is important to consider that NSCLC is a genomically highly unstable tumor type. Gene copy number gain of larger and focal chromosomal regions is present over the whole cancer genome of NSCLC and is also connected to a higher gene expression [39]. Our gene expression analysis revealed that *JAK2* is upregulated in PD-L1 amplified cases, and might act alone or in concert as an oncogenic driver [7, 27]. Therefore, it is possible that PD-L1 amplification is only a bystander event or surrogate marker of chromosomal instability in NSCLC. In particular, in region 9p24.1, several genes are considered to have tumorigenic potential; therefore, it cannot be excluded that PD-L1 amplification occurs as a random event associated with a general higher rate of genomic aberrations. This random nature, however, does not exclude that PD-L1 amplification plays an important role in the process of tumorigenesis, e.g., by an influence on the immune microenvironment. The associations in SqCC and the lack of significant associations in AC indicate that there might be more signals impacting cancer immunity in one way or another, which is different between these biologically distinct entities.

In conclusion, our study characterized PD-L1 amplification in a large, well annotated, NSCLC cohort. We found that PD-L1 amplification had a stronger molecular imprint in SqCC with an immune cell rich phenotype. We believe our study provides more evidence to further evaluate PD-L1 amplification as a predictive biomarker of immunotherapy.

## Availability of data and material

The datasets used and/or analyzed during the current study are available from the corresponding author upon reasonable request.

### Supplementary Information

Below is the link to the electronic supplementary material.

Supplementary file1 (PDF 6395 KB)

## Data Availability

All R packages used in this study are stated under Material and Methods.
