# Supplementary material for: PD-L1 amplification is associated with an immune cell rich phenotype in squamous cell cancer of the lung
Source: Cancer Immunol Immunother. 2021 Feb 12;70(9):2577–87. doi: 10.1007/s00262-020-02825-z (PMC8360842; doi:10.1007/s00262-020-02825-z)
Supplement: Supplementary file 1 — Supplementary file1 (PDF 6395 KB) [file 262_2020_2825_MOESM1_ESM.pdf]

Supplementary Material

PD-L1 amplification is associated with an immune cell rich phenotype in squamous cell cancer of the lung.

Goldmann T, Marwitz S, Nitschkowski D, Krupar R, Backman M, Elfving H, Thurfjell V, Lindberg A, Brunnström H, La Fleur L, Mezheyeuski A, Mattsson JSM, Botling J, Micke P, Strell C

Page 2 **Supplementary Table 1**: Association between PD-L1 amplification and clinico-pathologic parameters within Uppsala 06 and 95 cohort.

Page 3 **Supplementary Table 2**: Cox proportional hazards regression analysis for association of PD-L1 amplification status and 5-year overall survival within Uppsala 06 and 95 cohort.

Page 4 **Supplementary Table 3**: Differentially expressed genes of PD-L1 amplified cases within the adenocarcinoma subtype of the Uppsala 06 cohort.

Page 5 **Supplementary Table 4**: Differentially expressed genes of PD-L1 amplified cases within the squamous cell carcinoma subtype of the Uppsala 06 cohort.

Page 6 **Supplementary Figure 1:** FISH analysis of PD-L1 amplification.

Page 7 **Supplementary Figure 2:** Distribution of PD-L1 amplification, correlation with mRNA and protein within the Uppsala 95 cohort.

Page 8 **Supplementary Figure 3**: Correlation between the PD-L1 amplification ratio and PD-L1 mRNA and protein within the Uppsala 06 and 95 cohort.

Page 9/10 **Supplementary Figure 4:** Association of the PD-L1 amplification status with mutation status of 82 genes within the Uppsala 06 cohort.

Page 10 **Supplementary Figure 5:** Hierarchical cluster analysis using of IHC based immune marker annotation and association with PD-L1 amplification status and tumor histology in the Uppsala 95 cohort.

Page 11 **Supplementary Figure 6:** Hierarchical cluster analysis of immune exhaustion marker and HLA gene expression and association with PD-L1 amplification and tumor histology in the Uppsala 06 cohort.

Supplementary Tables

Supplementary table 1

Supplementary table 1: Association between PD-L1 amplification and clinico-pathologic parameters in Uppsala 06 and Uppsala 95 cohorts.

	Uppsala 06 cohort									Uppsala 95 cohort		
	Total (n=294)			AC (n=187)			SqCC (n=87)			Total (n=221)		
Variable	No PDL1 Amp	PDL1 Amp	p-value	No PDL1 Amp	PDL1 Amp	p-value	No PDL1 Amp	PDL1 Amp	p-value	No PDL1 Amp	PDL1 Amp	p-value
n (%)	280	14	-	179	8	-	81	6	-	211	10	-
Age, years:												
≤70	175 (62.5)	9 (64.3)	1.000	118 (65.9)	5 (62.5)	1.000	47 (58)	4 (66.7)	1.000	146 (69.2)	8 (80)	0.727
>70	105 (37.5)	5 (35.7)	-	61 (34.1)	3 (37.5)	-	34 (42)	2 (33.3)	-	65 (30.8)	2 (20)	-
Gender:												
female	144 (51.4)	8 (57.1)	0.787	100 (55.9)	6 (75)	0.469	36 (44.4)	2 (33.3)	0.692	94 (44.5)	7 (70)	0.192
male	136 (48.6)	6 (42.9)	-	79 (44.1)	2 (25)	-	45 (55.6)	4 (66.7)	-	117 (55.5)	3 (30)	-
Stage (TNM):												
Ia-Ib	175 (62.5)	10 (71.5)	0.582	112 (62.6)	6 (75)	0.712	52 (64.2)	4 (66.7)	1.000	149 (70.6)	6 (60)	0.490
IIa-IV	105 (37.5)	4 (37.5)	-	67 (37.4)	2 (25)	-	29 (35.8)	2 (33.3)	-	62 (29.4)	4 (40)	-
WHO performance score:												
0	175 (62.5)	10 (71.4)	0.582	117 (65.4)	6 (75)	0.717	45 (55.6)	4 (66.7)	0.692	113 (53.6)	3 (30)	0.198
1-3	105 (37.5)	4 (28.6)	-	62 (34.6)	2 (25)	-	36 (44.4)	2 (33.3)	-	98 (46.4)	7 (70)	-
Smoker:												
never	33 (11.8)	0 (0)	0.380	21 (11.7)	0 (0)	0.187	5 (6.2)	0 (0)	1.000	19 (9.1)	0 (0)	1.000
former/current	247 (88.2)	14 (100)	-	158 (88.3)	8 (100)	-	76 (93.8)	6 (100)	-	190 (90.9)	10 (100)	-
Histology:												
AC	179 (63.9)	8 (57.1)	0.769	-	-	-	-	-	-	115 (54.5)	3 (30)	**0.021**
SqCC	81 (28.9)	6 (42.9)	-	-	-	-	-	-	-	77 (36.5)	3 (30)	-
AdSq	5 (1.8)	0 (0)	-	-	-	-	-	-	-	-	-	-
LCC	5 (1.8)	0 (0)	-	-	-	-	-	-	-	19 (9)	4 (40)	-
LCNEC	8 (2.9)	0 (0)	-	-	-	-	-	-	-	-	-	-
SC	2 (0.7)	0 (0)	-	-	-	-	-	-	-	-	-	-

Abbreviations: Amp = amplification; WHO = world health organization; AC = adenocarcinoma; SqCC = squamous-cell carcinoma; AdSq = adenosquamous carcinoma; LCC = large cell carcinoma; LCNEC = large cell neuroendocrine carcinoma; SC = sarcomatoid carcinoma

* p-values are based on Fisher’s exact test, two-sided

Supplementary table 2

Supplementary table 2:
Cox proportional hazards regression analysis for association of PD-L1 amplification status and 5-year overall survival in Uppsala 06 and Uppsala 95 cohorts.

	Uppsala 06 cohort						Uppsala 95 cohort	
	Total (n=310)		AC (n=196)		SqCC (n=94)		Total (n=224)	
Variable	HR (95% CI)	p-value*	HR (95% CI)	p-value*	HR (95% CI)	p-value*	HR (95% CI)	p-value*
PD-L1 status, UVA								
wt	1	-	1	-	1	-	1	-
amplified	0.62 (0.25-1.51)	0.289	0.63 (0.20-1.99)	0.430	0.65 (0.16-2.71)	0.557	1.64 (0.80-3.36)	0.176
polysomy	0.88 (0.43-1.80)	0.730	0.82 (0.33-2.02)	0.668	1.03 (0.32-3.35)	0.962	1.14 (0.28-4.63)	0.851
PD-L1 status, MVA								
wt	1	-	1	-	1	-	1	-
amplified	0.65 (0.27-1.59)	0.344	0.77 (0.24-2.46)	0.659	0.60 (0.14-2.51)	0.481	1.94 (0.93-4.04)	0.076
polysomy	0.73 (0.36-1.51)	0.403	0.74 (0.30-1.83)	0.507	0.69 (0.20-2.33)	0.546	0.95 (0.23-3.85)	0.940
Age, years:								
≤70	1	-	1	-	1	-	1	-
>70	0.91 (0.65-1.27)	0.569	0.94 (0.62-1.42)	0.754	0.59 (0.30-1.16)	0.125	1.17 (0.80-1.71)	0.428
Gender:								
female	1	-	1	-	1	-	1	-
male	1.37 (0.99-1.91)	0.058	1.21 (0.80-1.83)	0.372	1.61 (0.82-3.14)	0.166	1.37 (0.94-2.01)	0.104
Stage (TNM):								
Ia-Ib	1	-	1	-	1	-	1	-
IIa-IV	2.08 (1.5-2.89)	<0.001	2.64 (1.74-4.00)	<0.001	1.55 (0.82-2.93)	0.181	2.20 (1.51-3.22)	<0.001
WHO performance score:								
0	1	-	1	-	1	-	1	-
1-3	1.38 (0.81-2.54)	0.222	1.44 (0.96-2.12)	0.082	1.81 (0.94-3.49)	0.078	1.94 (1.34-2.81)	<0.001
Smoker:								
never	1	-	1	-	1	-	1	-
Former/current	1.41 (1.01-1.96)	0.043	3.01 (1.25-7.65)	0.015	0.37 (0.11-1.28)	0.117	1.55 (0.66-3.65)	0.321

Abbreviations: HR = hazard ratio; CI = confidence interval; UVA = univariable analysis; MVA = multivariable analysis; AC = adenocarcinoma; SqCC = squamous-cell carcinoma; WHO = world health organization

* p-values are based on Wald test

Supplementary table 3:

Differentially expressed genes (false discovery rate p-adjusted <0.05) of PD-L1 amplified cases within the adenocarcinoma subtype of the Uppsala 06 cohort.

SYMBOL	GENENAME	Base Mean	log2 Fold Change	p-value	p-adj
ETNPPL	ethanolamine-phosphate phospho-lyase	69,23	-23,84	0,0000	0,0000
DSCAM-AS1	DSCAM antisense RNA 1	109,10	-24,48	0,0000	0,0000
FABP7	fatty acid binding protein 7	229,50	7,57	0,0000	0,0000
SNX31	sorting nexin 31	20,21	4,90	0,0000	0,0000
CPS1	carbamoyl-phosphate synthase 1	14534,93	-5,69	0,0000	0,0020
SSC4D	scavenger receptor cysteine rich family member with 4 domains	84,79	2,52	0,0000	0,0020
MUC13	mucin 13, cell surface associated	2802,00	-5,43	0,0000	0,0036
TESC	tescalcin	1851,99	-3,60	0,0000	0,0061
COL25A1	collagen type XXV alpha 1 chain	596,33	-4,89	0,0000	0,0099
PAH	phenylalanine hydroxylase	305,05	-5,03	0,0000	0,0101
ARL14	ADP ribosylation factor like GTPase 14	124,99	-4,37	0,0000	0,0101
STARD3	StAR related lipid transfer domain containing 3	2199,24	0,99	0,0000	0,0107
SMS	spermine synthase	1721,00	1,13	0,0000	0,0133
ASCL1	achaete-scute family bHLH transcription factor 1	420,63	-6,39	0,0000	0,0133
LRAT	lecithin retinol acyltransferase	205,16	-2,57	0,0000	0,0140
CALCA	calcitonin related polypeptide alpha	1304,42	-6,81	0,0000	0,0153
CTNND2	catenin delta 2	475,09	-4,82	0,0000	0,0153
BARX1	BARX homeobox 1	147,18	-6,24	0,0000	0,0161
TFPI2	tissue factor pathway inhibitor 2	6597,47	-3,78	0,0000	0,0178
FGB	fibrinogen beta chain	3677,79	-6,56	0,0000	0,0178
GK5	glycerol kinase 5	1637,82	1,17	0,0000	0,0178
SYT13	synaptotagmin 13	583,13	-4,98	0,0000	0,0197
LINC00319	long intergenic non-protein coding RNA 319	89,19	-2,70	0,0000	0,0239
CHGB	chromogranin B	663,52	-4,52	0,0000	0,0329
NOL4	nucleolar protein 4	59,24	-4,35	0,0000	0,0335
NCKAP5-AS2	NCKAP5 antisense RNA 2	19,05	-3,72	0,0000	0,0460
ZP1	zona pellucida glycoprotein 1	15,10	2,60	0,0001	0,0481

Supplementary table 4:

Differentially expressed genes (false discovery rate p-adjusted <0.05) of PD-L1 amplified cases within the squamous cell carcinoma subtype of the Uppsala 06.

SYMBOL	GENENAME	Base Mean	log2 Fold Change	p-value	p-adj
RCL1	RNA terminal phosphate cyclase like 1	1440,42	2,14	0,0000	0,0000
SOX1	SRY-box transcription factor 1	30,60	-22,21	0,0000	0,0000
PLPP6	phospholipid phosphatase 6	919,97	2,17	0,0000	0,0000
DLK1	delta like non-canonical Notch ligand 1	56,07	-22,81	0,0000	0,0000
AK3	adenylate kinase 3	5296,48	1,85	0,0000	0,0000
CDC37L1	cell division cycle 37 like 1	1413,59	1,76	0,0000	0,0000
GLDC	glycine decarboxylase	613,14	4,91	0,0000	0,0000
RIC1	RIC1 homolog, RAB6A GEF complex partner 1	4138,46	1,47	0,0000	0,0000
PRB4	proline rich protein BstNI subfamily 4	24,42	-21,90	0,0000	0,0000
PLGRKT	plasminogen receptor with a C-terminal lysine	1174,06	1,76	0,0000	0,0000
PUM3	pumilio RNA binding family member 3	3300,58	1,59	0,0000	0,0000
KDM4C	lysine demethylase 4C	2544,03	1,37	0,0000	0,0001
DMAC1	distal membrane arm assembly complex 1	926,28	1,39	0,0000	0,0001
LINC00665	long intergenic non-protein coding RNA 665	1058,57	-2,59	0,0000	0,0001
UHRF2	ubiquitin like with PHD and ring finger domains 2	4813,49	1,20	0,0000	0,0004
RANBP6	RAN binding protein 6	2949,62	1,48	0,0000	0,0005
OTOF	otoferlin	44,25	2,98	0,0000	0,0008
SPATA6L	spermatogenesis associated 6 like	236,22	1,86	0,0000	0,0009
SNORD11	small nucleolar RNA, C/D box 11	9,11	2,52	0,0000	0,0012
CSDE1	cold shock domain containing E1	42738,74	-0,87	0,0000	0,0038
SCIN	scinderin	837,52	2,84	0,0000	0,0044
ZNF829	zinc finger protein 829	282,86	-1,82	0,0000	0,0044
ZNF703	zinc finger protein 703	1101,67	-1,91	0,0000	0,0163
KIAA2026	KIAA2026	3345,84	1,09	0,0000	0,0183
ARHGEF5	Rho guanine nucleotide exchange factor 5	1100,41	1,30	0,0000	0,0190
THEGL	theg spermatid protein like	5,98	-5,05	0,0000	0,0191
JAK2	Janus kinase 2	3061,45	1,39	0,0000	0,0211
ZNF212	zinc finger protein 212	929,60	0,85	0,0000	0,0252
ZNF420	zinc finger protein 420	578,81	-1,17	0,0000	0,0253
ZFP30	ZFP30 zinc finger protein	741,18	-1,11	0,0000	0,0269
TACR1	tachykinin receptor 1	88,66	3,10	0,0000	0,0294
CD274	CD274 molecule	3337,90	2,30	0,0000	0,0326
ZNF568	zinc finger protein 568	441,23	-1,28	0,0001	0,0340
SULT1E1	sulfotransferase family 1E member 1	528,12	-4,35	0,0001	0,0344
CXCL5	C-X-C motif chemokine ligand 5	1157,49	-2,89	0,0001	0,0344
LINC00863	long intergenic non-protein coding RNA 863	81,26	-1,27	0,0001	0,0375
IGFL3	IGF like family member 3	40,23	-5,34	0,0001	0,0461
NGF	nerve growth factor	36,70	-2,85	0,0001	0,0484
IL33	interleukin 33	4349,46	2,26	0,0001	0,0491
AMTN	amelotin	2641,54	-5,71	0,0001	0,0491

Supplementary Figures

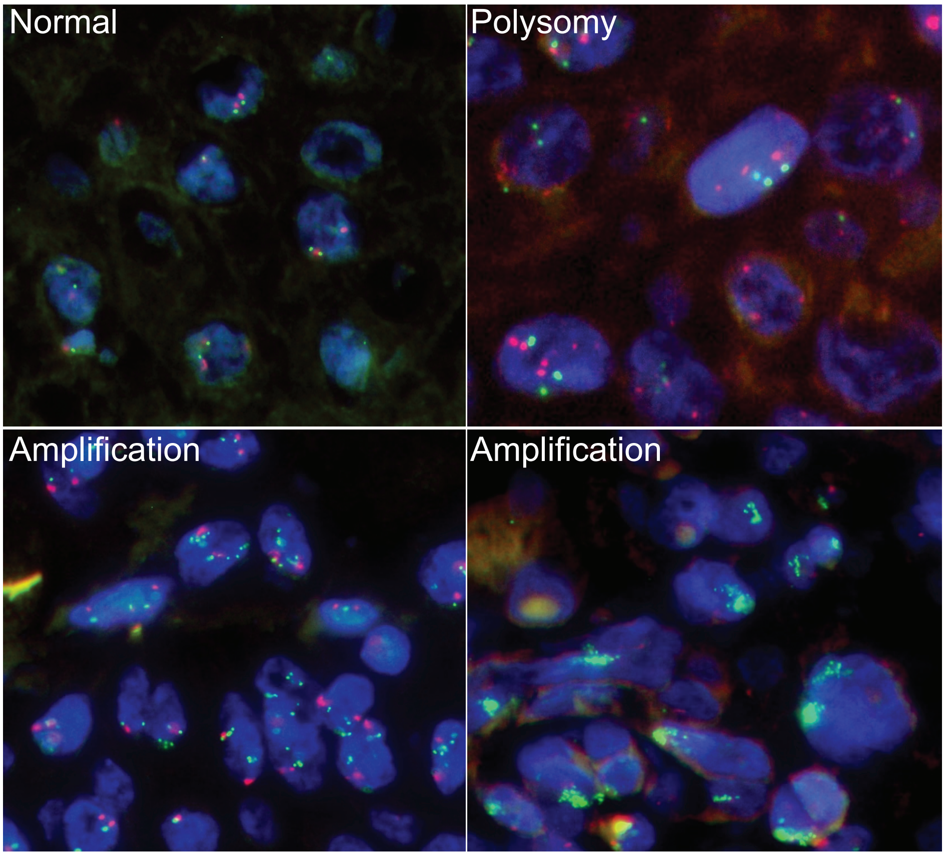

Supplementary Figure 1: Example images of FISH analysis for PD-L1 amplification. In normal interphase nuclei, two orange (D9Z3 at 9q12) and two green signals (CD274,PDCD1LG2 gene cluster at 9p24.1) are detected. In cells with amplification of the CD274,PDCD1LG2 gene cluster, multiple copies of the green signal or large green signal clusters are observed. Cases were defined as polysome with PD-L1 gene copy number gain when the ratios of PD-L1/ centrosome 9 were 1 and there were more than two copies of PD-L1 and centrosome 9 within a single tumor cell. PD-L1 was considered as amplified, when the ratios of PD-L1 signals to centrosome 9 signals in the tumor cells were ≥ 2.

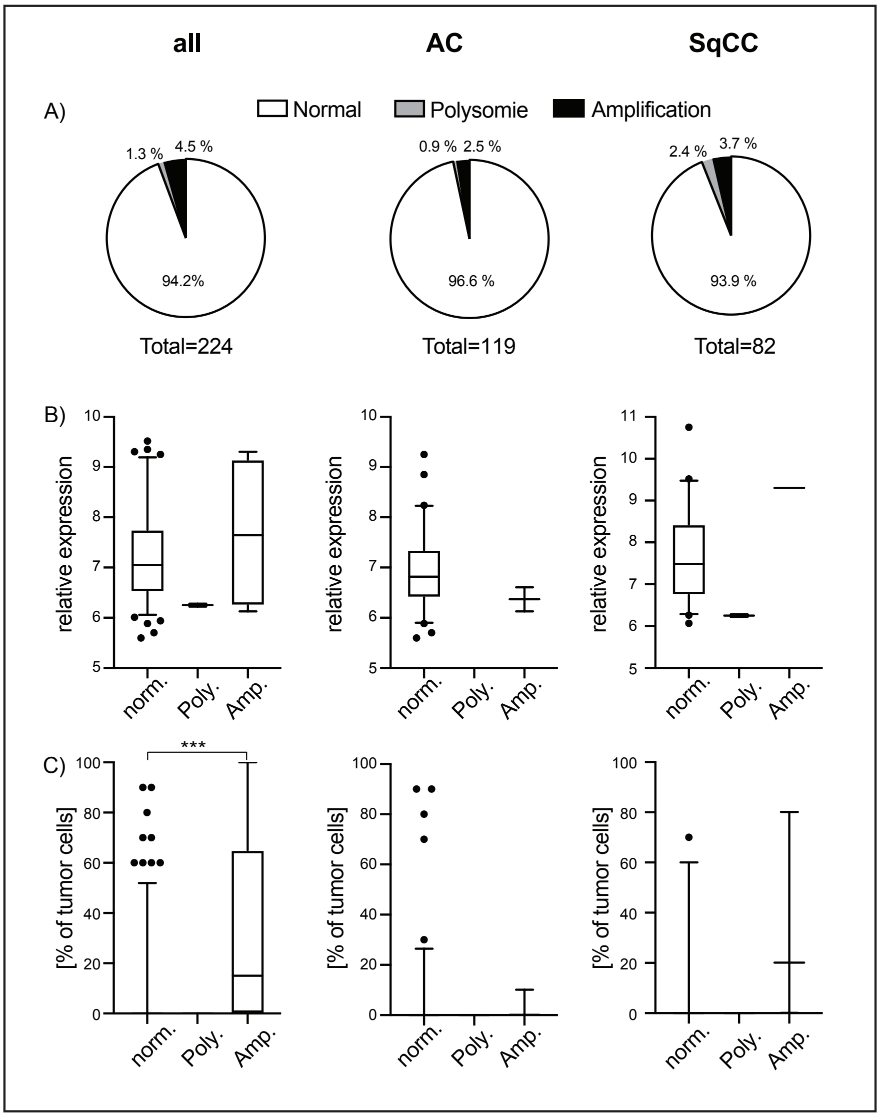

Supplementary Figure 2: (A) Percentual distribution of PD-L1 amplified cases and cases with PD-L1 polysomy among all included NSCLC patients as well as separately within the adenocarcinoma (AC) and squamous cell carcinoma (SqCC) tumor subgroups of the Uppsala 95 cohort. (B and C) Association of the PD-L1 amplification status with (B) PD-L1 mRNA expression (array-based) and (C) percentage of PD-L-positive tumor cells on protein level as determined by IHC. Box plots indicate the median with the interquartile range and the whiskers represent the 5-95 percentiles. Outliners are represented as dots. P-values are based on Kruskal-Wallis test (two-sided) with Dunn's correction for multiple testing. *** p<0.001

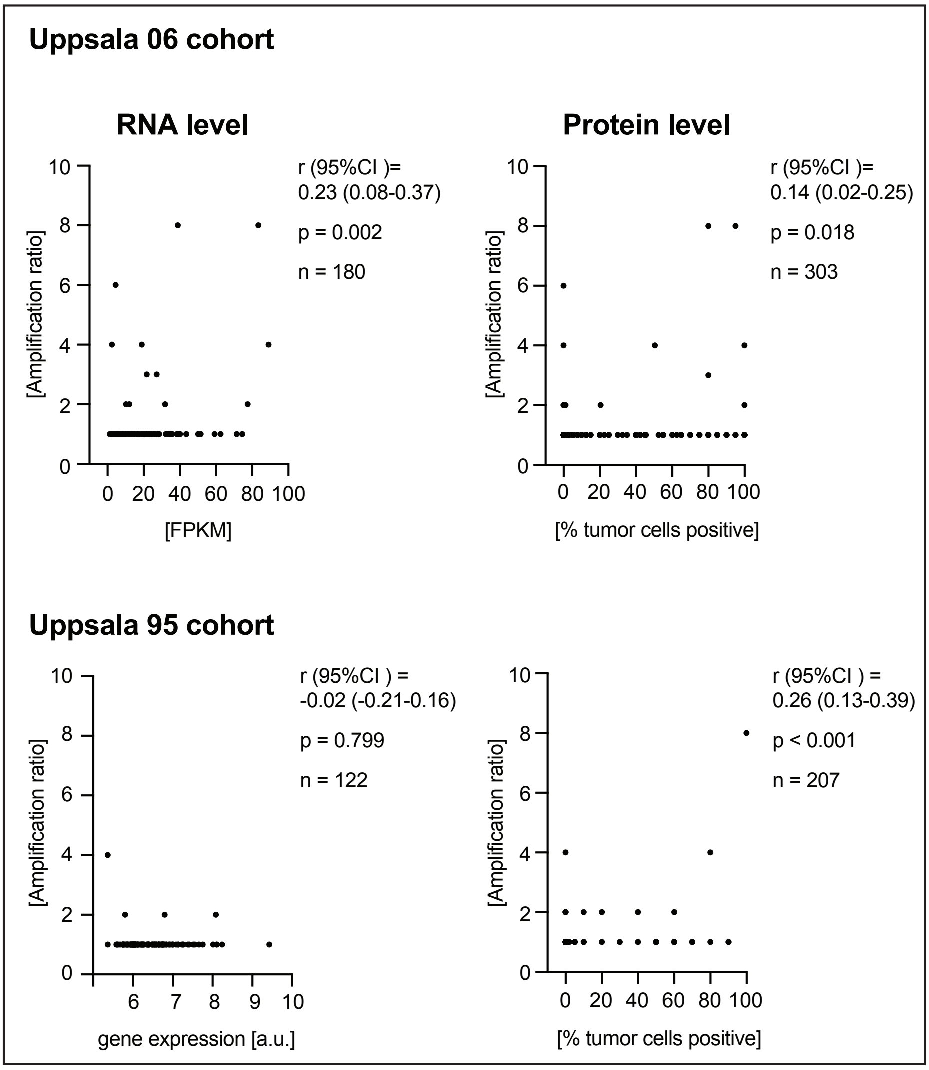

Supplementary Figure 3: Correlation between the PD-L1 amplification ratio and PD-L1 mRNA expression (as FPKM for Uppsala 06 cohort and as relative expression in abbreviated units for Uppsala 95 cohort) and percentage of PD-L-positive tumor cells on protein level as determined by IHC. Spearman's rank correlation coefficient r with 95% confidence interval (CI) and two-sided p-value are stated as well as the number of samples included in the test (n).

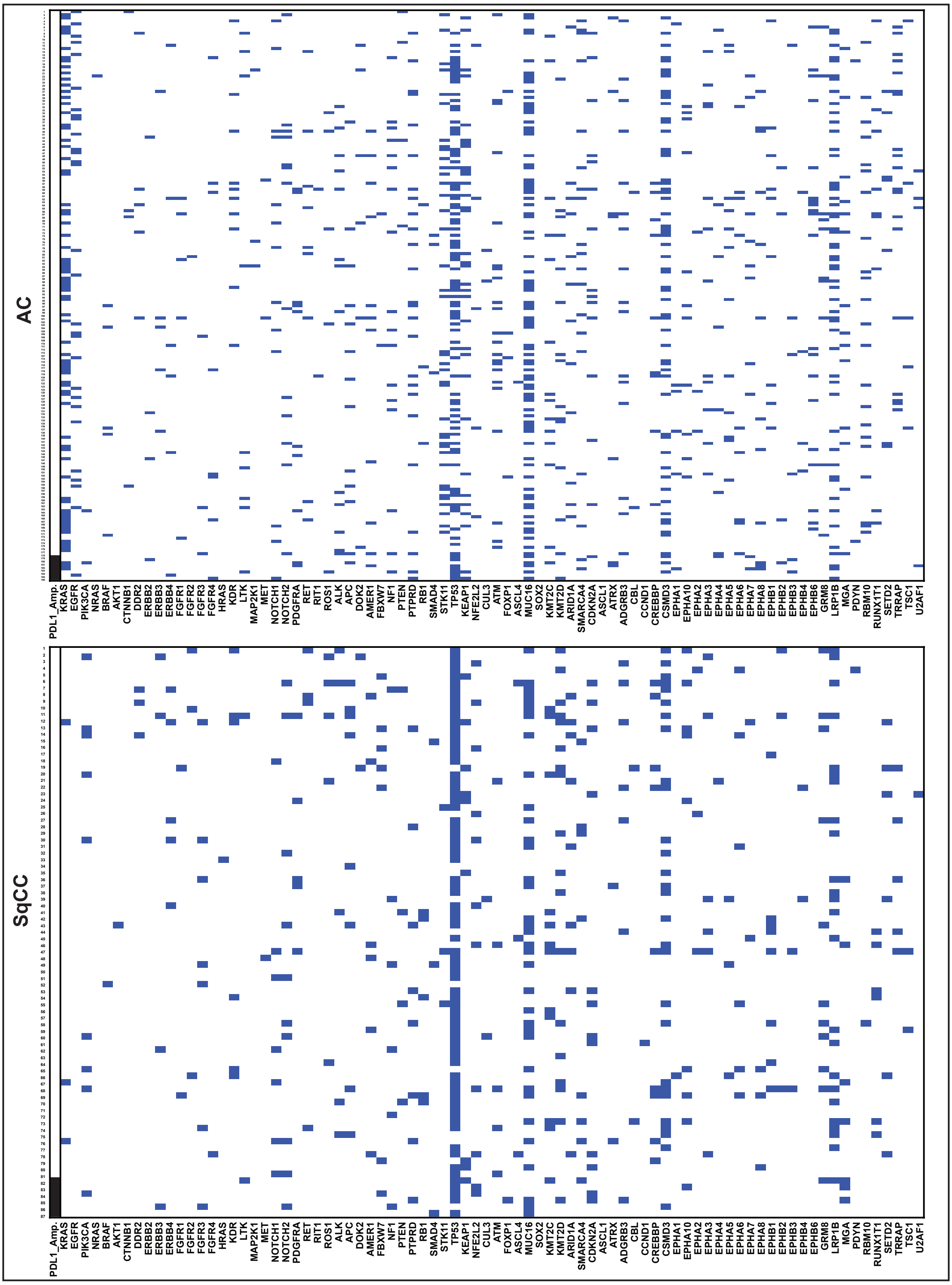

Supplementary Figure 4: Uppsala 06 cohort; Association of the PD-L1 amplification status (black) with mutation status of 82 genes (blue) represented as binary heat map for adenocarcinoma (AC) and squamous-cell carcinoma (SqCC) tumor subgroups separately. For

the *KRAS, EGFR, PIK3CA, NRAS, BRAF, ERBB2* and *MET* genes only activating driver mutations were considered in the analysis.

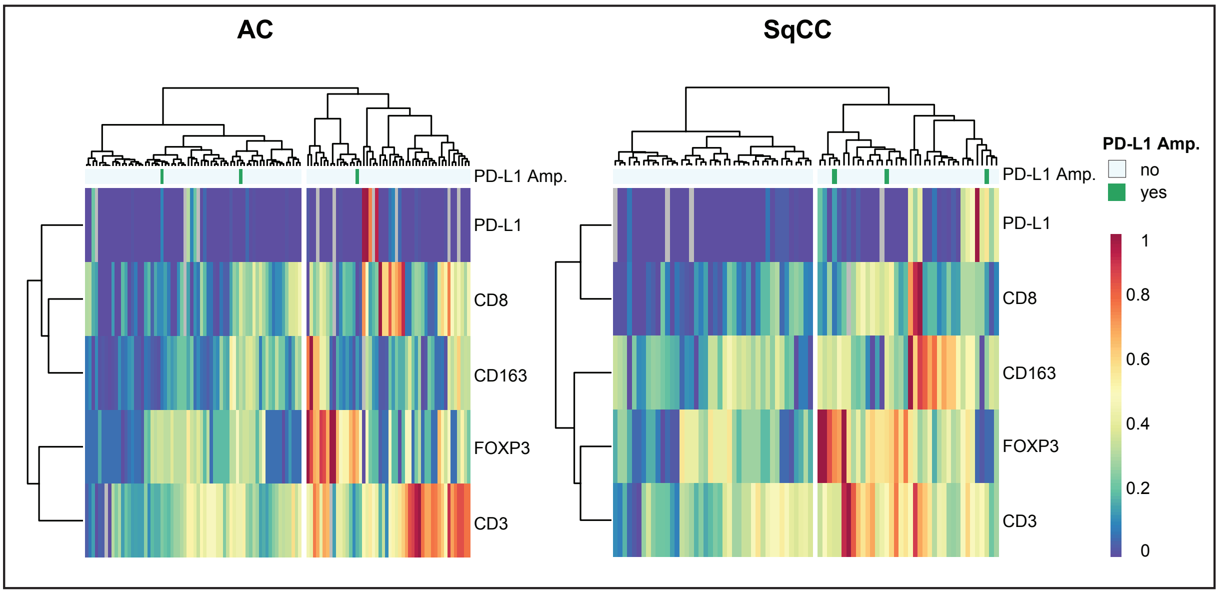

Supplementary Figure 5: Hierarchical cluster analysis using Ward's method and Euclidian distance of the IHC based immune marker annotation and its association with PD-L1 amplification status within the adenocarcinoma (AC) and squamous cell carcinoma (SqCC) tumor subgroups of the Uppsala 95 cohort.

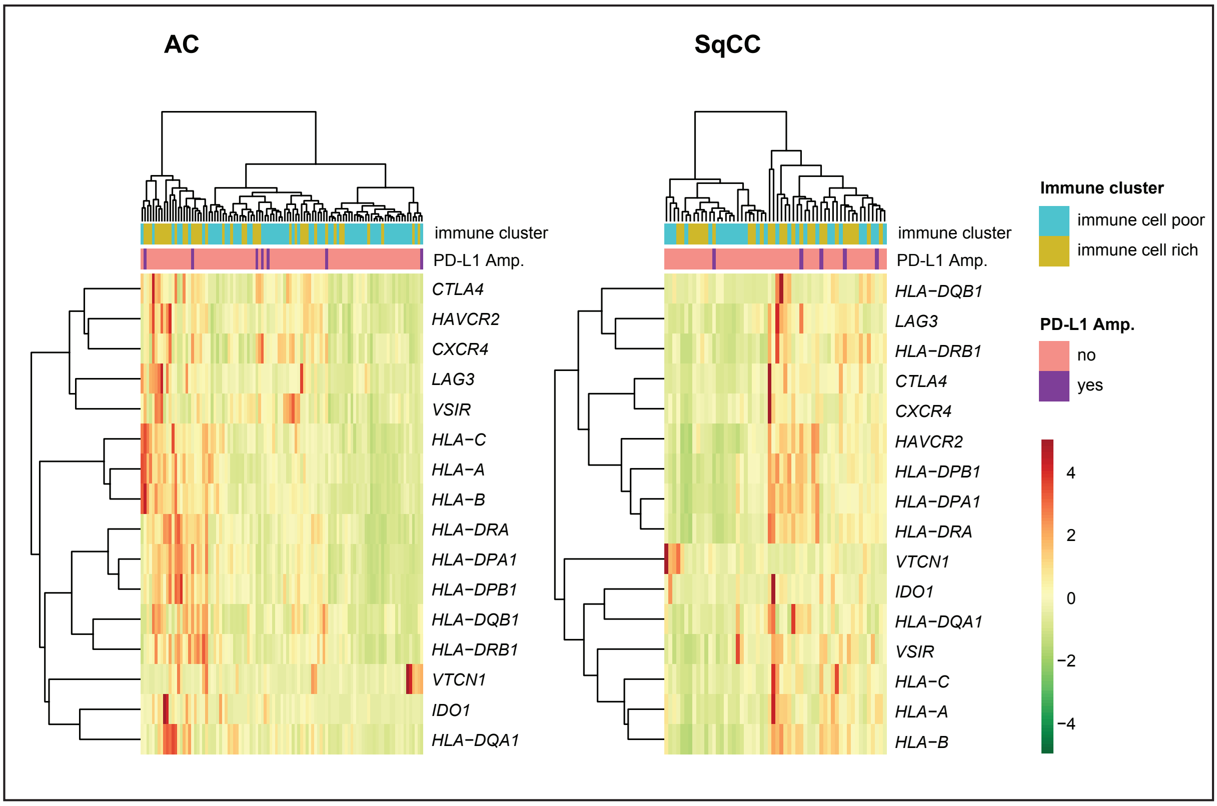

Supplementary Figure 6: Hierarchical cluster analysis using Ward's method and Euclidian distance of genes related to immune exhaustion as well as HLA expression and their association with PD-L1 amplification status, immune cluster and tumor histology (AC = adenocarcinoma; SqCC = squamous cell carcinoma) within the Uppsala 06 cohort. Immune clusters are defined based on main Figure 3.
